# Surgical management of ostomy complications: a MISSTO–WSES mapping review

**DOI:** 10.1186/s13017-023-00516-5

**Published:** 2023-10-10

**Authors:** Dario Parini, Andrea Bondurri, Francesco Ferrara, Gianluca Rizzo, Francesco Pata, Marco Veltri, Cristiana Forni, Federico Coccolini, Walt L. Biffl, Massimo Sartelli, Yoram Kluger, Luca Ansaloni, Ernest Moore, Fausto Catena, Piergiorgio Danelli, Multidisciplinary Italian Study group for STOmas (MISSTO)

**Affiliations:** 1 General Surgery Department, Santa Maria della Misericordia Hospital, Rovigo, Italy; 2 General Surgery Department, Luigi Sacco University Hospital, ASST Fatebenefratelli-Sacco, Milano, Italy; 3 Department of Surgical, Oncological and Stomatological Sciences, University of Palermo, Palermo, Italy; 4 Digestive and Colorectal Unit, Fatebenefratelli Isola Tiberina Gemelli Isola Hospital, Università Cattolica del Sacro Cuore, Roma, Italy; 5 Department of Pharmacy, Health and Nutricional Sciences, University of Calabria, Cosenza, Italy; 6 Department of Surgery, Nicola Giannettasio Hospital, Corigliano-Rossano, Italy; 7 General Surgery Unit, San Jacopo Hospital, Pistoia, Italy; 8 Nursing and allied profession research Unit, IRCCS Istituto Ortopedico Rizzoli, Bologna, Italy; 9 General, Emergency and Trauma Surgery Department, Pisa University Hospital, Pisa, Italy; 10 Trauma/Acute Care Surgery, Scripps Clinic Medical Group, La Jolla, CA USA; 11 General Surgery Department, Macerata Hospital, Macerata, Italy; 12 Division of General Surgery, Rambam Health Care Campus, Haifa, Israel; 13 General Surgery Department, Pavia University Hospital, Pavia, Italy; 14 E. Moore Shock and Trauma Centre, Denver, CO USA; 15 General, Emergency and Trauma Surgery Department, Bufalini Hospital, Cesena, Italy; 16 Department of Biomedical and Clinical Sciences “Luigi Sacco”, University of Milan, Milano, Italy

**Keywords:** Stoma complication, Surgical management, Review, Stoma necrosis, Mucocutaneous separation, Stoma retraction, Stoma prolapse, Parastomal hernia, Stoma stenosis, Stoma bleeding

## Abstract

**Background:**

The creation of an ileostomy or colostomy is a common surgical event, both in elective and in emergency context. The main aim of stoma creation is to prevent postoperative complications, such as the anastomotic leak. However, stoma-related complications can also occur and their morbidity is not negligible, with a rate from 20 to 70%. Most stomal complications are managed conservatively, but, when this approach is not resolutive, surgical treatment becomes necessary. The aim of this mapping review is to get a comprehensive overview on the incidence, the risk factors, and the management of the main early and late ostomy complications: stoma necrosis, mucocutaneous separation, stoma retraction, stoma prolapse, parastomal hernia, stoma stenosis, and stoma bleeding.

**Material and methods:**

A complete literature research in principal databases (PUBMED, EMBASE, SCOPUS and COCHRANE) was performed by Multidisciplinary Italian Study group for STOmas (MISSTO) for each topic, with no language restriction and limited to the years 2011–2021. An international expert panel, from MISSTO and World Society of Emergency Surgery (WSES), subsequently reviewed the different issues, endorsed the project, and approved the final manuscript.

**Conclusion:**

Stoma-related complications are common and require a step-up management, from conservative stoma care to surgical stoma revision. A study of literature evidence in clinical practice for stoma creation and an improved management of stoma-related complications could significantly increase the quality of life of patients with ostomy. Solid evidence from the literature about the correct management is lacking, and an international consensus is needed to draw up new guidelines on this subject.

**Supplementary Information:**

The online version contains supplementary material available at 10.1186/s13017-023-00516-5.

## Introduction

The creation of a temporary or permanent stoma (colostomy or ileostomy) is indicated in several pathological conditions, such as malignant (colorectal cancer) or benign conditions (inflammatory bowel disease, diverticular disease, intestinal obstruction or perforation, trauma). The main aim of stoma creation is to improve the quality of life, allowing the treatment of the pathological condition or preventing the occurrence and the negative consequences of postoperative complications, such as the anastomotic leak [1]. In 2019, the Multidisciplinary Italian Study group for STOmas (MISSTO) published the guidelines for the surgical management of stomas [2], which described the key steps of the preoperative and intraoperative management of ostomy creation, with the purpose of preventing or reducing the occurrence of stoma-related complications and underlying the most frequent risk factors for stoma-related morbidity [3]. The latter can develop as a result of surgical- or patient-related factors (Table 1). In particular, emergency stomas have shown to have a higher complication rate: a recent study [4] showed a higher rate of complications in the emergency cohort with a significant rate for Clavier-Dindo’s grade 3 (6% vs 20%) and 4 (1% vs 8%). The overall rate of postoperative stoma-related complications is between 20 and 70% [5–8]. This range cannot be considered as negligible, according to the annual rate of stoma creation in the USA (more than 100,000) or in the UK (more than 20,000) [9]. Stoma-related postoperative complications, which can occur within 30 days (early complications) or after several months from stoma creation (late complications), adversely impact the quality of life of stoma patients and significantly increase the costs of the health service [10].

**Table 1:** Risk factors associated with stoma-related complications (from Babakhanlou R., Larkin K., Hita A.G.et al. Stoma-related complications and emergencies.Int J Emerg Med 2022;15:17)

Patient-related factors	Medical and surgical risk factors
Cardiac comorbidities	Emergency surgery
Respiratory comorbidities	Surgery for malignancy
Musculoskeletal comorbidities	Poor surgical technique
Diabetes	Surgeon’s experience and specialty
Smoking	No preoperative input from a stoma nurse
Cancer	Concomitant chemotherapy
Obesity (BMI > 30)	Corticosteroid therapy
Age (> 60 years)	Preoperative radiation
Poor nutritional status	

Most stomal complications are managed non-operatively, through a multidisciplinary and multi-specialist approach. MISSTO in 2021 published the guidelines for the nursing management of stoma-related morbidity [11]. In this context, a key role is played by nurses specialized in stoma care. Sometimes, the conservative approach is not effective to solve the complication, and surgical treatment becomes necessary. The aim of this mapping review is to get a comprehensive overview on the incidence, the risk factors, and the management (conservative and surgical) of the main surgical complications of ostomies, identifying research gaps and collecting evidence for future research directions.

## Methods

The approach used for the elaboration of this mapping review followed four steps. The first step included the formulation of the clinical questions. This was based on the previous experience of the working group that elaborated clinical practice guidelines for the management of ostomies [2]. The working group decided to focus the aim of the review on a topic that was not previously extensively investigated: the management of surgical complications of ileostomy and colostomy.

The second step was the research of relevant evidence, performed by literature search of studies from January 2011 to December 2021, with no language restriction, on this topic. After careful literature evaluation, the research group focused the study on the most frequent stoma complications needing surgical treatment, also in emergency setting: stoma necrosis, mucocutaneous separation, stoma retraction, stoma prolapse, parastomal hernia, stoma stenosis, and stoma bleeding [12]. Another important complication is dehydration with possible electrolyte abnormalities, especially in patients with ileostomy. Its management is often clinical and only in selected cases surgical resolution with early closure is possible [13]. This complication has not been evaluated in the present review, and it should need a separate study. The research was performed on PubMed, EMBASE, and Cochrane database of systematic reviews, and Scopus, and included every type of article. Exclusion criteria were studies reporting on pediatric patients (< 18 years of age), studies reporting on gastro-/oesophago- or duodenostomies as well as urostomies, studies with unclear work-up, and studies focused only on the prevention of stoma complications.

The third step included literature evaluation. When available, the most recent guidelines, systematic reviews and meta-analysis, were considered as the best evidence for their extensive literature review, including additional resources retrieved from references in analyzed articles. Then, randomized clinical trials were also considered, and, if not available, other study types, including non-randomized studies, quasi-experimental studies, cohort studies, case–control studies, descriptive studies, expert consensus, and expert opinions, were evaluated for extraction of relevant data on each specific topic. Two authors independently screened all titles and abstracts of papers identified by the search strategies for relevance. They only excluded clearly irrelevant reports at this stage. We obtained full copies of all potentially relevant papers. Afterward, the two review authors independently screened the full texts, identified relevant studies, and assessed eligibility of studies for inclusion. They resolved disagreements on the eligibility of studies by discussion and consensus, or if necessary, by consulting a third review author.

In the last step, the authors elaborated summary of the best available evidence regarding the topics of the study each of which was classified according to: definition, incidence, classification, and treatment.

Manuscript was finally reviewed and revised by an international panel from MISSTO and the World Society of Emergency Surgery (WSES).

Detailed study protocol methods and results are available in the appendix (Additional file 1).

### Stoma complications

1. Stoma necrosis

#### Definition, epidemiology, and classification

Necrosis of the stoma is a significant early complication that results from an inadequate blood supply, and it is divided into superficial (necrosis of the bowel mucosa) and deep (necrosis beyond the mucosa of the bowel) [14].

Definitive diagnosis between congestion and necrosis is crucial and time demanding: newly constructed stoma appears edematous and cyanotic in the immediate postoperative period but as postoperative edema decreases, the stoma usually shrinks [15]. The causes of necrosis are associated with the surgical technique in stoma creation, including tension on the mesentery, ligation of the primary blood vessel, excessive dissection of the peristomal mesentery and constriction in the abdominal wall due to excessively small opening in the fascia, abdominal wall mesh, or skin [1, 13, 16].

The overall incidence of stoma necrosis ranges between 1.6% up to 20% [13, 14, 17–21].

Stoma necrosis usually occurs during the few days after surgery but can present in the first postoperative months. A retrospective study on 144 patients observed end-colostomy necrosis on the ward in 14 of 80 patients (17.5%) and in 3 of 66 patients (4.5%) at 3 months [22].

Specific risk factors for stoma necrosis include emergent operations, colostomies, obesity, the use of a rod for lateral ostomy, and surgery performed by non-colorectal surgeons [4, 15, 22]. The obese patient is seven times more likely to experience stoma necrosis than the non-obese patient. Stoma necrosis is much less common for loop: in an observational study on 84 patients with lateral ileostomy the incidence of necrosis was 2.3% [16].

End-colostomies are at risk of necrosis: in a retrospective study exploring complications in standard abdominoperineal resection vs extra-elevator resection for rectal cancer, the incidence of colostomy necrosis was 35% in patients operated on in the prone jackknife position, significantly higher than in patients operated with standard procedure (13 of 38 vs. 3 of 32, p < 0.05) [23].

Intracutaneous suturing of the ileostomy was not found to be superior to transcutaneous suturing with regard to stomal complications, including necrosis (1.8% vs 3.6%), in the ISI trial [24].

The placement of a rod in constructing lateral ostomy (using a dedicated plastic rod or a part of drain or catheter) is often correlated to local necrosis [25]. Two RCT’s study reported a higher incidence of necrosis in participants managed by rods [17, 19]. In contrast, a prospective cohort study reported a higher mean incidence of stoma necrosis, but the difference was not statistically significant [18]. Another systematic review on 1004 patients concluded that there was a higher rate of stoma necrosis (rod 7% vs no rod 1.15% OR 5.58; 95% CI 1.85–16.84) in the rod group [26] (Table 2).

**Table 2:** Summary of treatments for stoma necrosis

Type of treatment	Indications	Drawbacks
Non-operative management	Superficial or small deep necrosis	Risk of mucocutaneous separation Risk of stenosis Patient discomfort
Emergency surgical treatment	Deep necrosis (below the fascia) with signs of intra-abdominal contamination	Risk of surgical and non-surgical complications
Elective surgical treatment	Deep necrosis (above the fascia)	Risk of surgical and non-surgical complications

#### Non-operative management

The decision to proceed with stoma revision depends on the level of stoma necrosis in the abdominal wall [1]. The extent of ischemic changes in the mucosa can be effectively assessed using flexible (pediatric) endoscopy through the stoma site or using a proctoscope or a clear test tube or a combination of standard video bronchoscope inserted into a clear plastic blood collection tube [13, 15, 27].

If the necrosis is superficial, there is no need for revision [15]. Gentle debridement and conservative management can safely be considered, although this management strategy can ultimately result in longer-term complications such as retraction or stenosis [13]. If the length of necrosis is more than 1 or 2 cm, early revision could be considered to prevent future stenosis stoma revision, although it is important to note that stoma revision is technically much easier when early intense inflammatory adhesions and bowel and mesentery edema have subsided [15].

A cross-sectional quantitative study with narrative-type components was recently used to identify optimal interventions for selected complications, including necrosis, based on ostomy nurse expertise. Respondents reported that the management of stomal necrosis would include the use of a transparent, two-piece pouching system to allow stoma access, use of a lubricated clear test tube to check level of necrosis, and referral for debridement of necrotic tissue as necessary. Use of a nitroglycerine patch or peristomal ointment was noted as not evidence-based [28].

In selected cases, it is possible to treat superficial necrosis with negative pressure wound therapy (NPWT) by isolating the stoma and treating the peristomal wound area [29].

As with all stomal complications, necrosis results in delayed hospital discharge, increased community stoma care, and delay in the initiation of adjuvant chemotherapy for colorectal cancer patients [21].

Ostomy nurse telephone follow-up is effective to enhance postoperative adjustment of early discharged colostomy patients even in case of necrosis diagnosed at the baseline [30].

#### Surgical management

If the necrosis extends below the fascia, an immediate surgery is required with resection of the ischemic bowel. The extent of the bowel resection depends upon the extent of necrosis and ischemia and ultimately on the ability of the bowel conduit to reach the skin level. The surgeon must be prepared to create a new stoma at a new site [15] or, if indicated, to close the stoma. Intense inflammatory adhesions, together with bowel and mesentery edema, can make stoma revision or closing difficult during the first few postoperative weeks.

2. Mucocutaneous separation

#### Definition, epidemiology, and classification

Mucocutaneous separation (MCS) is defined as a partial (partial MCS) or circumferential (complete MCS) detachment of the mucosa from the peristomal skin at various levels of deep (superficial, if it involves only epidermidis; deep, if it involves dermis and subcutaneous layers) with or without an associated abscess [1, 31, 32].

MCS is reported as the most frequent ostomy-related complication with an incidence higher than 15% [33, 34]. In a large prospective study on 1,427 stoma patients, the rate of overall ostomy-related complications was 38.8% and the most frequent complication was MCS with a rate of 18.6% [33]. This rate of MCS incidence was confirmed in another large study (retrospective) on 462 stoma patients in which the rate of MCS was 19.5%.

The presence of an ileostomy seems to represent a risk factor for the occurrence of a MCS; in this type of stoma, the risk of MCS is described in a range between 8.5% and 20.5% [33, 35, 36], higher than MCS colostomy rate (3–4%) [37]. In the group of ileostomies, the rate of MCS seems to be influenced also by the height of the distal limb of the stoma from the skin. In a large retrospective multicenter study on 4137 patients with ileostomy, the rate of MCS was higher in the group with a height of the distal limb < 1 cm (11.2 vs 2.9% with a height of distal limb > 1 cm) [32].

Conditions that increase the tension on the stoma or favor stoma necrosis increase the risk of MCS [10, 38–40]. In this context, as recently reported by a Chinese systematic review and meta-analysis, a demonstrated risk factor for the occurrence of a MCS is stoma support rods, which seems to double the risk of MCS if compared with ostomy created without using rod. An alternative option to standard rods, without significantly increasing the rate of MCS, is skin bridge which seems to be associated with a rate of MCS significantly lower than traditional rods [41].

Factors hindering the healing process such as infectious, malnutrition, diabetes mellitus, and chronic immunosuppressive therapy such steroids significantly increase the risk of MCS [38, 40, 42].

For stomas created in an IBD setting, the use of immunosuppressive drugs (such as vedolizumab) in the preoperative period seems to significantly (sixfold) increase the risk of MCS if compared to stoma patients who did not receive vedolizumab preoperatively, also including patients preoperatively treated with anti-TNF therapy [31, 43] (Table 3).

**Table 3:** Summary of treatments for mucocutaneous separation (MCS)

Type of treatment	Indications	Drawbacks
Non-operative management	Superficial or small deep MCS	Risk of infections Risk of enlargement of MCS Patient discomfort
Emergency surgical treatment	Deep MCS with signs of intra-abdominal contamination	Risk of surgical complications (infections, recurrence, ischemia) Risk of non-surgical complications
Elective surgical treatment	Superficial MCS after failure of conservative treatment because: circumferential MCS sign of severe infections severely symptomatic patients Deep MCS without signs of intra-abdominal contamination	Risk of surgical and non-surgical complications

#### Non-operative management

The conservative management of MCS is possible for superficial or small deep MCS. The conservative management of superficial MCS include several actions such as irrigation with isotonic saline solutions, covering of the area with absorbent or insulating products, and a more frequent replacement of the stoma device. The concomitant presence of infection, malnutrition, or other systemic causes of MCS need a correction with the help of a specialist, even when superficial, circumferential MCS needs close observation to prevent progression to deep MCS. In the presence of deep MCS, it may be effective to fill the gap using alginate or gelling fiber and cover it with a solid hydrocolloid or the pouch’s skin barrier [1, 38, 44]. In mildly symptomatic patients, a convex appliance may be useful to decrease bowel leakage [45]. If infection occurs, an antimicrobial dressing may be useful in addition to systemic antibiotic therapy [44].

#### Surgical management

Surgical management of MCS became necessary in deep MCS and in superficial MCS after failure of conservative treatment. Stoma revision by local repair, with partial mobilization of the proximal bowel, can be attempted, but the definitive treatment is stoma reversal, when possible, or stoma relocation by a laparotomy in a site and on a loop which secures an adequate bowel length and blood supply [11]. With this assumption, a new local approach based on the placement of a Dracon vascular prothesis was described by Feres et al. [45]. This technique consists of anastomosing a segment of approximately 5 cm of DVP at the edge of an intestinal fistula with an absorbable monofilament 4–0 thread in a continuous way, with anchors placed at the beginning and half of the suture. Theoretically, the healing process should promote the adhesion of the prosthesis to the stoma, preventing the escape of secretions to the peristomal skin, and the collection bag is properly attached to receive the effluent [45].

3. Stoma retraction

#### Definition, epidemiology, and classification

Stoma retraction (SR) is commonly defined as a condition in which the stoma mucosa is more than 0,5 cm below the skin level; the term retraction usually also includes conditions in which the stoma pulls the surrounding skin inward (due to excessive bowel tension) or in which the stoma is within a skin fold (especially in a sitting position) [32, 46]. To establish a condition of SR, it is necessary to respect two criteria: dimensional (stoma must be 0.5 cm or more below the skin surface) and temporal (it appears within 6 weeks from stoma creation) [14, 47–49]. Temporal criteria were also used to classify the SR in early (within 30 days from surgery) and late (beyond 30 days after surgery) [48].

A large retrospective analysis on 462 ostomy patients reported a 3.2% incidence of SR, the second most frequent stoma-related complication after mucocutaneous separation [34].

It is not clear if the type of stoma, ileo- or colostomy, influences the rate of SR [47, 50]. In a systematic review and meta-analysis by Rondelli et al., there was no significant difference about the incidence of SR in ileostomy or colostomy patients (respectively 1.6% vs 3.1%) [50]. On the other hand, a prospective study by Robertson et al. reported a significantly higher incidence of SR in colostomy group (22% vs 8% in ileostomy group) during the first 10 postoperative days, but this difference was leveled at 2 years from stoma surgery (13% vs 11%) [47].

The stoma conformation seems to influence the SR rate. The creation of an end ileostomy seems to expose patients to a higher rate of SR. Van der Sluis et al. reported a rate of SR of 10% after creation of an end ileostomy [51], significantly higher than the incidence recorded in other studies analyzing loop ileostomy, which reported a range of 1.3–3% [52, 53]. The differences in terms of SR between end- or loop- stoma configuration were reported also in colostomy patients, where the rate of SR seems to be higher after a loop colostomy (13.9% vs 0.9–4% with end colostomy) [54, 55].

The use of a rod (in loop stomas) does not seem to reduce the rate of SR. In a review of 1131 stoma patients, the rate of SR in the rod group was not significantly lower than the rate recorded in patients without rod (2.3% vs 3.4%) [41, 48].

SR seems to be more frequent in patients with a high body mass index (BMI), due to the difficulty in mobilization and exteriorization of a thickened mesentery [49].

The presence of an inflammatory bowel disease (IBD) represents a risk factor for SR, due to the long-term use of steroid and/or immunosuppressive drugs and to the thickened and fibrosis of the mesentery secondary to the inflammatory disease, especially in Crohn’s disease patients. Indeed, Crohn’s disease is considered a risk factor for SR as reported by the study of Takahashi et al., in which the rate of SR in Crohn’s disease was significantly higher than the rate recorded in ulcerative colitis patients (6.6% vs 2.2%) [56].

Other reported risk factors for SR are excessive tension on the stoma, leading to ischemia and necrosis of the stoma, inadequate mobilization of the bowel, inadequate positioning of the stoma itself, aggressive postoperative fluid resuscitation, long-term steroid use, malnutrition, diabetes, smoking, poor wound healing, and peristomal infection [14, 16, 41, 47, 48, 51, 57].

The occurrence of an early SR (within 30 days from stoma creation) is more frequently due to an inadequate or difficult mobilization of intestinal loop, to large adipose tissue or to a stoma creation in a non-suitable site. High BMI and chronic peristomal dermatitis (with secondary scar tissue) represent the more frequent causes of a late SR [15] (Table 4).

**Table 4:** Summary of treatments for stoma retraction

Type of treatment	Indications	Drawbacks
Non-operative management	Limited and superficial stoma retraction (intestine at the skin or at subcutaneous level)	Risk of deeper retraction Patient discomfort
Emergency surgical treatment	Deep stoma retraction with signs of intra-abdominal contamination	Risk of surgical complications (infections, recurrence, ischemia) Risk of non-surgical complications
Elective surgical treatment	Failure of conservative approach to allow an adequate adhesion of stoma appliances Persistent complications, especially infectious and skin complications Deep stoma retraction without intra-abdominal contamination	Risk of surgical and non-surgical complications

#### Non-operative management

If the bowel retraction is limited and superficial (intestinal loop at the skin or at subcutaneous level), a conservative management is possible, but a daily stoma examination is mandatory to guarantee an early diagnosis of a deeper retraction. As reported in the recent Italian guidelines on stoma care, the conservative management of a SR should include the use of a convex or flexible stoma appliance and the use of belt and other accessories (ring or hydrocolloidal strips) to increase the convexity and the adherence on the skin of stoma appliances [11].

#### Surgical management

Surgical revision of a retracted stoma should be considered in case of: failure of conservative approaches to allow an adequate adhesion of stoma appliances; persistent complications, especially infectious and skin complications, frequently due to a not adequate adhesion of the stoma appliances; deeper stoma retraction and risk of peritoneal contamination by stools [11].

Because the excessive tension of the bowel, primary cause of SR, is the result of an inadequate mobilization of the intestinal loop, the rationale of surgical management of SR is based on the increase in the stoma length by dissection of the bowel [15]. This result can be achieved with a local approach or, if inadequate, by a midline laparotomy. The local repair of SR is a procedure, which is performed through the stoma site and is based on the partial mobilization of the proximal bowel. However, the efficacy of the local repair depends on the level of the retraction and is possible only if the SR is due to superficial factors and not to intra-abdominal factors.

In the context of local repair of a SR, Skærlund et al. described a technique of the retracted stoma by a non-cutting linear stapler, which could be performed also in outpatient setting, and is based both on an intestinal superficial mobilization and on a stoma fixation to the skin. In this technique, the stoma is grasped with two Babcock clamps and everted at approximately 3 cm above skin level; between the two clamps, a non-cutting linear stapler is fired from the tip of the stoma to its base at skin level; two more staples are then fired around the circumference. Even if this technique could include additional sessions, the rate of recurrence of SR is about 50% [58–61].

If local approaches failed in the treatment of SR, because the underlying and intra-abdominal causes of tension cannot be solved through a peristomal incision, a laparotomy approach should be considered. The treatment of SR by laparotomy consists in remaking the stoma using a longer and more mobile bowel loop, which guarantees the creation of a stoma without traction and tension. If the laparotomic correction of SR is needed, it is also advisable to reallocate the stoma in a new abdominal site, different from the previous one, in order to avoid the possible traction effect of skin scar reaction [10, 15]. Finally, stoma reversal, when possible, is the best surgical solution.

4. Stoma prolapse

#### Definition, epidemiology, and classification

Stoma prolapse is a late complication of stoma formation and is defined as a full-thickness telescoping protrusion of bowel through a stoma. Prolapse is more common with colostomies than ileostomies, above all with loop transverse colostomies, where most frequently involves the distal limb [13, 15, 37, 62, 63]. Risk factors for stoma prolapse include advanced age, obesity, abdominal wall laxity, a large fascial opening, redundant or mobile bowel proximal to the stoma (in particular in Hartmann procedure), bowel obstruction at the time of stoma creation, and postoperative conditions increasing intra-abdominal pressure, as chronic cough, ascites, or constipation. Absence of preoperative site marking is an additional risk factor [5]. This late complication has an incidence between 2 and 26% [13, 64]. Stoma prolapse can be classified as sliding, if it occurs intermittently with increased intra-abdominal pressure, or fixed, if it is present constantly (Table 5).

**Table 5:** Summary of treatments for stoma prolapse

Type of treatment	Indications	Drawbacks
Non-operative management	Sliding prolapse Fixed prolapse in high risk patients	Persistence of prolapse Patient discomfort Risk of complications
Emergency surgical treatment	Bowel occlusion Prolapse incarceration/strangulation Stoma gangrene	Risk of recurrence High rate of surgical complications High need of laparotomic approach
Elective surgical treatment	Parastomal hernia associated Failure of non-operative management of sliding prolapse Fixed prolapse without complications	Risk of surgical complications More complex surgery

#### Non-operative management

Conservative management of stoma prolapse first should consider modification of the pouching system, for reducing friction between bowel prolapsed mucosa and the sac with lubricants, manual reduction of prolapse and use of a hernia support belt to restrain the stoma loop. Patient and caregiver should be educated to recognize signs and symptoms of complications, in particular ischemia, necrosis and bowel strangulation.

The management of non-reducible stomal prolapse includes the reduction of edema by the application of cold or soaked tablets of hypertonic glucose solutions or direct application of sucrose [11].

#### Surgical treatment

The most common complaint that leads to elective repair is inability to manage conservatively the problems with ostomy care, where the appliance does not adhere to the skin and fails to respond to conservative measures, further subjecting the prolapsed intestine to reiterative trauma and bleeding [65]. Emergency surgery is required when strangulation, ischemia, or necrosis of the bowel prolapsed occur. Bowel obstruction consequent to stoma prolapse is another indication to surgical repair, often in an emergency setting.

Theoretically, the best surgical treatment for stoma prolapse is stoma reversal, but often this procedure is not yet indicated or definitively not possible, if stoma is considered as permanent. The surgical technique to repair stoma prolapse is surgeon dependent and not standardized. In general, there are two types of approach: a local repair and an abdominal access, via laparotomy or laparoscopy. The simplest local repair technique involves incision of the mucocutaneous junction, dissection into the peritoneal cavity, reduction to the abdominal cavity of the redundant bowel and refashioning of the stoma at the same site [66]. An alternative local technique for lateral stoma is to perform, after mucocutaneous separation, the transection of loop stoma, obtaining two separated bowel segments. The proximal one is used to realize an end-loop stoma, and the distal can be matured as a mucus fistula. When the prolapsed bowel appears ischemic, it is recommended to resect it before refashioning the stoma [15]. The abdominal approach is indicated when there’s a suspicion of peritoneal cavity contamination, a necessity of additional bowel mobilization or to re-siting the stoma. Common technique involves a midline laparotomy, or a laparoscopic approach. Laparoscopy is possible not only in elective surgery, but even in emergency setting, with stable patient and in expert hands. Loop bowel prolapsed is reduced through the abdominal wall aperture and then is fixed with sutures to the anterior abdominal wall, in an “accordion-like” fashion. If it is indicated to resect prolapsed bowel, it is necessary to realize a mucocutaneous separation before loop reduction. If the fascial aperture is too large, it is possible to reduce it with a direct suture or, if there is an associated parastomal hernia, to reinforce the abdominal wall with mesh [66].

Re-siting of the stoma is indicated when the previous stoma site is not reusable, due to a skin or wall infection or an excessive dimension of the wall aperture.

In general, local surgical solution allows local or spinal anesthesia, while abdominal approach requires general anesthesia. It is important to consider this aspect when patient general conditions are compromised.

5. Parastomal hernia

#### Definition, epidemiology, and classification

Parastomal hernia (PSH) is an incisional hernia associated with a stoma, resulting in a protrusion of abdominal content through the defect in the abdominal wall created during the creation of a colostomy, ileostomy, or ileal conduit [2, 67, 68]. For the purposes of this study, we will not discuss PSH associated with urinary stomas.

The incidence of PSH is variable depending on the definition, diagnostic method or classification system used, depending also on the follow-up of the studies. Most reports describe incidence rates greater than 30%, reaching higher rates for colostomies, as high as 86% [67, 69, 70].

Different diagnostic modalities have been described in the literature. The first and most direct method is physical examination, with observation of a protrusion over the abdominal wall around the stoma, especially if the patient is performing a Valsalva maneuver in erect or supine position. However, this method is affected by poor inter-observer reliability [67, 68, 70]. For this reason, an imaging technique, such as abdominal ultrasound (US) or computed tomography (CT scan), is often used to confirm the clinical diagnosis. The difference in the detection rates of PSH between clinical examination and CT scan is unknown [68, 69]. Moreover, this diagnostic method is not without inaccuracies; in fact, it may fail to detect PSH in 7% of cases. Differences in the detection reliability of CT scan may exist if the examination is performed in the supine or prone position or during Valsalva maneuver [68]. Although no gold standard exists for the diagnostic method of PSH, CT scan is universally accepted as the technique of choice to confirm the diagnosis or obtain better characterization of parastomal hernia [67, 68], with a sensitivity of 83% compared to clinical examination alone [70] (Table 6).

**Table 6:** Classification systems of PSH

System	Types	Description
EHS (radiological and intraoperative)	Type I	PSH defect area ≤ 5 cm without concomitant incisional hernia
	Type II	PSH defect area ≤ 5 cm with concomitant incisional hernia
	Type III	PSH defect area > 5 cm without concomitant incisional hernia
	Type IV	PSH defect area > 5 cm with concomitant incisional hernia
Moreno-Matias (radiological)	Type 0	Normal, peritoneum follows the wall of the bowel forming the stoma with no formation of a sac
	Type Ia	Bowel forming the colostomy with a sac < 5 cm
	Type Ib	Bowel forming the colostomy with a sac > 5 cm
	Type II	Sac containing omentum
	Type III	Intestinal loop other than the bowel forming the stoma
Devlin (intraoperative)	Type I	Interstitial hernia
	Type II	Subcutaneous hernia
	Type III	Intrastomal hernia
	Type IV	Peristomal hernia (stoma prolapse)
Rubin (intraoperative)	Type Ia	True parastomal interstitial hernia
	Type Ib	True parastomal subcutaneous hernia
	Type II	Intrastomal hernia
	Type III	Subcutaneous prolapse
	Type IV	Pseudohernia (connected with flank insufficiency or denervation)
Gil and Szcepkowski (clinical)	Type I	Isolated small parastomal hernia
	Type II	Small parastomal hernia with coexisting midline incisional hernia
	Type III	Isolated large parastomal hernia
	Type IV	Large parastomal hernia with coexisting midline incisional hernia

Several classification systems have been proposed, based on 3 examination methods: physical examination, intraoperative findings, or radiological description [69, 71]. Devlin et al. proposed an anatomical classification of PSH with 4 subgroups: interstitial, subcutaneous, intrastomal, or peristomal depending on the location of the hernia sac [72]. Rubin and Bailey described a similar anatomical classification system with four types of PSH: true parastomal hernia, intrastomal, subcutaneous prolapse, and pseudohernia [73].

Gil and colleagues proposed another classification based on structural criteria. They proposed four groups: type I includes small, isolated parastomal hernias, without coexisting cicatricial hernia, and without anterior abdominal wall deformation; type II involves parastomal hernias with associated cicatricial hernia, without considerable deformation of the abdominal wall; type III includes large, isolated parastomal hernias without coexisting cicatricial hernia; type IV includes large parastomal hernias with coexisting cicatricial hernia [74].

However, these anatomical classifications are of limited clinical value; in fact, they have never been used in clinical or research settings. So radiological classification systems have been proposed to standardize evaluation between studies and to help planning surgical treatment.

Moreno-Matias (MM) and colleagues proposed a radiological classification system based on the detection at CT scan of the hernia sac content and size, with strong correlation to the clinical examination. In this system, type 0 is described as the normal situation in which the peritoneum follows the wall of the bowel forming the stoma, with no formation of a sac; in the type Ia, the hernial sac contains the loop forming the stoma, with sac diameter < 5 cm, it is considered a prehernial stage; in the type Ib, the hernial sac contains the loop forming the stoma, with sac diameter > 5 cm; in the type II, the hernial sac contains the omentum; in the type III, the hernial sac contains a loop of bowel different to that forming the stoma [75]. The authors demonstrated that clinical detection of PSH increases with the progression to type Ia to type III and other authors also demonstrated that all type III PSH patients experienced symptoms [76]. The European Hernia Society (EHS) proposed another classification to create a homogeneous system to improve the ability to compare different studies and cohorts of patients. The authors included variables regarding symptoms, the choice of treatment and treatment prognosis, and they proposed a classification system based on the size of PSH and the presence of coexistent incisional hernia (cIH). Subclasses of classification were defined as follows: type I PSH < 5 cm without cIH; type II PSH < 5 cm with cIH; type III PSH > 5 cm without cIH; type IV PSH > 5 cm with cIH (P: primary PSH; R: recurrence after previous PSH treatment). Although CT scans could be performed preoperatively and could help determine the subgroup of the defect, the authors recommend intraoperative measurement procedure [68].

However, for both the MM and EHS classification systems the relationship between PSH classification and the need for surgical repair has not been evaluated. Frigault and colleagues, in a retrospective cohort study, investigated the correlation between the classification systems, the radiological findings and the need for surgical repair of PSH. In their study, they classified all radiological positive patients for PSH according to both MM and EHS systems. MM type III and EHS type I were the most common PSH observed in the study (53% and 63%, respectively). No correlation was shown between the MM classification and the need for surgical repair, and between these classifications and radiological signs of small-bowel obstruction at the stoma site. Moreover, the authors did not evaluate a potential correlation with clinical diagnosis because radiological assessment was not always followed by clinical evaluation of the stoma. The authors concluded that the EHS classification correlated with the need for surgical repair during follow-up [67] (Table 7).

**Table 7:** Summary of treatments for PSH

Type of treatment	Indications	Drawbacks
Non-operative management	PSH with no complications Patient preference	Risk of future complications
Emergency surgical treatment	Strangulation Incarceration Obstruction	Risk of surgical complications: recurrence, infections Risk of non-surgical complications
Elective surgical treatment	Patient discomfort Other complications not requiring emergency surgery: i.e. skin complications, prolapse	Risk of surgical and non-surgical complications

#### Non-operative management

There is scarce evidence about the need of treating PSH and non-operative management seems to be appropriate in most cases [69, 77]. Watchful waiting is a common practice for patients with PSH, since the decision of surgical repair is tailored to patient preference after considering all potential surgical risks against the possibility of future hernia complications [69, 78]. Some authors suggest consulting an enterostomal therapy nurse for patients and surgeons choosing non-operative management for assistance in the case of appliance leakage or fitting a hernia holder or belt [71].

The main benefit of this approach is the absence of the risks of complications and recurrence associated with the surgical repair in patients that usually report mild or lack of symptoms. On the other hand, this strategy may lead to some complications, such as strangulation, enlargement of the defect and development of comorbidities, which may increase the risks of subsequent surgical repair [77, 78]. In a retrospective study of 80 patients with PSH, the main reasons for choosing non-operative management were absence of complaints (32%) and presence of comorbidities (24%) [77].

The EHS guidelines on PSH management conclude that, in light of little evidence in the literature, no recommendation on the watchful waiting strategy can be made [78].

#### Surgical treatment

Although PSH can be managed non-operatively in most cases, about 30% of these patients may ultimately require surgical repair [79], due to the onset of complications, such as strangulation, incarceration or obstruction, or to discomfort and problems with the fitting and function of the appliance [75]. Of these, 10–20% may need urgent surgery. Indications for emergent surgical intervention include severe abdominal pain, nausea, vomiting, and constipation associated with incarcerated or strangulated PSH [69].

Other indications for surgical repair are mainly patient-based, and they depend on the reported symptoms and impairment. These may include bulge around the stoma, poorly fitting appliance, discomfort at site, and recurrent symptoms of partial bowel obstruction [69, 78].

When the stoma is temporary, ostomy closure may be an optimal alternative to PSH repair, although the risks associated with stoma reversal may be considered together with about 30% risk of developing incisional hernia in the site of closure with simple fascial suture of the defect [79].

Simple fascial repair is associated with an elevated risk of recurrence. A meta-analysis described a recurrence rate of 69.4% if it is performed locally or through midline laparotomy [80]. In the EHS guidelines, the authors conclude that, although the high recurrence rate, this technique may be considered in specific patient groups, such as in contaminated cases or in the emergency repair of a strangulated hernia, for the less risks compared to the use of a mesh and because this is the simplest surgical repair technique [78].

Another option is stoma relocation, but it is associated with high recurrence rate at the new stoma site, as high as 76% in some studies, along with the risk of ventral hernia formation at the site of the previous ostomy [69, 79].

PSH mesh repair can be performed with an open or minimally invasive approach (laparoscopic, endoscopic or robotic). No randomized controlled trials have been performed comparing different methods of PSH repair, and the literature consists primarily of single institution series with relatively small numbers describing outcomes using one type of repair and subsequent reviews of these series.

The reported advantage of mesh repair compared to simple fascial closure is the lower recurrence rate (5–15%). Moreover, the fear of mesh infection has proved to be unfounded, with reported rates of 2–3% and surgical site infection rates of about 4%, lower than primary suture repair [80]. Thus, mesh repair is considered the most effective option in PSH surgical treatment.

Meshes can be placed in different anatomic positions: onlay, retromuscular, or intraperitoneal. Although there are no randomized controlled trials comparing these techniques, the onlay placement has been associated with higher recurrence rates (15–17%) compared with the sublay or intraperitoneal positions (7–10%) [81].

The Sugarbaker technique, first published in 1985, is the most common used for PSH repair, consisting in securing a ring of prosthetic mesh in an underlay fashion deep to the fascial defect, and lateralizing the bowel immediately proximal to the stoma exit with a small gap to allow the intestine to pass through the mesh which is positioned intraperitoneal [82]. In the modified Sugarbaker technique, the fascial defect and lateralized colon are covered with mesh, making sure there is an overlap of at least 5 cm in all directions [79].

Another technique frequently used is the intraperitoneal keyhole, with placement of a mesh with a central hole or a slit around the bowel loop forming the stoma. Other techniques include the sandwich, inverted top hat, and stapled transabdominal ostomy reinforcement with retromuscular mesh [2, 69, 78].

Meshes can be also placed in the retromuscular space, by open or minimally invasive technique. This approach avoids intraperitoneal placement of mesh and the potential complications, as adhesions to the mesh, erosion or fistula formation [83].

Different mesh types can be used; however, there is scarce comparative evidence. Biologic meshes are associated with high recurrence rates (16–90%), and they can be considered only for selected cases, like in contaminated fields or as bridge options to definitive surgical repair. Synthetic meshes are considered the best options, with different materials, and with coated surfaces in the case of intraperitoneal technique. Several studies have reported the use of expanded-polytetrafluoroethylene (ePTFE) mesh for the minimally invasive or open intraperitoneal approaches. However, this type of mesh tends to shrink, so in most cases surgeons prefer to use composite mesh like ePTFE-polypropylene (PP), with ePTFE side faced to the viscera. Other types of mesh used in these cases are polyvinylidene fluoride (PVDF) mesh, composite PVDF-PP and dual-sided barrier-coated composite-type mesh. With these products, the coated side is placed in contact with the abdominal viscera to minimize bowel adhesions, and the superior surface is placed to optimize tissue incorporation into the abdominal wall. Lightweight and medium-weight PP meshes are instead used in both open or minimally invasive onlay and retromuscular techniques [78, 79].

Several systematic reviews, meta-analysis and guidelines have been produced about the management of PSH. However, studies are very heterogeneous as regards patient factors, surgical techniques, and types of mesh used and no consensus among authors still exists. There is no significant difference between the open and minimally invasive approaches nor in the specific technique type used for the repair. Sublay and intraperitoneal repair approaches have been found superior to onlay in terms of recurrence, with each approach demonstrating different complication profiles [81].

6. Stoma stenosis

#### Definition, epidemiology, and classification

Stoma stenosis is defined as an impairment between the stoma orifice and its output sufficient to cause a wide variety of symptoms, ranging from noisy bowel flatus, low-output stoma, abdominal distention to obstruction [2, 84]. Pragmatically, it has been defined as the impossibility to perform a digital examination or the introduction of a Hegar dilator n. 12 through the stoma orifice [85].

Stoma stenosis has been reported with different rates in the literature, ranging from 1 to 17% [14, 55, 86, 87]. This may reflect a difference in follow-up duration as well as in the definition of stoma complications. In a systematic review including 1009 patients from 18 randomized control trials reporting stoma-related complications as outcome, stomal stenosis was reported only in 6 studies, with a median incidence of 0.7%, 2.6% and 2.5% in loop-ileostomy, loop-colostomy, and end-colostomy group, respectively [12]. In a single-center study including 144 patients having an intestinal ostomy after urgent surgery with a two-year follow-up, stenosis was the most frequent complications along with retraction and separation in end colostomy, with no cases in the early postoperative period and the first cases reported at 3-month follow-up visit, with an overall rate of 11.63% [22]. In a single-center retrospective study, including 146 patients undergoing operation with a stoma creation with a mean follow-up of 28 months (range 3–183), stenosis occurred in only 0.7% (1 patient) [62].

Ambe et al. in their review of intestinal stomas report a stenosis rate of 2–17% in ileostomy and 1–14% in colostomy [87].

Stenosis may occur in the early postoperative period, as a technical failure due to inadequate stoma creation at skin or fascia level, but more commonly occurs later, weeks or months after the operation, isolated or as consequence of other postoperative complications, such as retraction, necrosis (with early ischemia as main contributing factor) [88] or after mucocutaneous separation, due to the wound contraction caused by the secondary healing [15, 88]. Preoperative radiotherapy, peristomal inflammation, and repeated microtrauma are other risk factors [11]. The repeated trauma related to ill-fitting pouching systems, with too large uncovered skin left around the stoma, has been reported as a causative factor [39, 89].

Stoma after surgery for Crohn disease may be more prone to stenosis due to mesenteric inflammation or adhesions for previous surgery, making bowel exteriorization more challenging. A recurrence of IBD may also play a role [65]. Rarely, a stomal stenosis may be secondary to a cancer arising in or near the stoma site in a long-standing permanent stoma [84].

Diagnosis of stomal stenosis is usually clinical, based on reduced output from stoma with symptoms ranging from abdominal distension, abdominal pain or discomfort up to intestinal occlusion in the severe form in a stoma orifice not or hardly explored by lubricated finger [2, 11]. An improvement of symptoms after stoma output may represent evidence to confirm the diagnosis. In case of impossible digital examination, a retrograde study by a small rubber catheter may confirm the diagnosis and inform about the length of the stenosis [39]. In a Crohn patient, an extensive preoperative diagnostic work-up is recommended to stage the disease before any operative decision [11] (Table 8).

**Table 8:** Summary of treatments for stoma stenosis

Type of treatment	Indications	Drawbacks
Non-operative management	Mild-moderate stenosis Bridge to surgery	Further fibrosis and stenosis Poor results in the long-term
Emergency surgical treatment	Rare indication: technical failure for inadequate procedure with early postoperative occlusion	Further surgical stress often in high-risk, fragile patients
Elective surgical treatment	Moderate or severe stenosis Stenosis not responding to non-operative management	Risk of high complications rate in case of laparotomy and stoma refashioning if local revision not suitable

#### Non-operative management

Conservative management represents the first option in the treatment of stomal stenosis. A low-residue diet with additional fluids possibly associated with stool softeners may be helpful in mild cases. In the end colostomies, irrigation may work as well [11, 85].

Progressive dilatation of the stoma orifice by Hegar dilators of increasing diameter across multiple sessions is the most solid option [15, 62, 84, 89]. Unfortunately, especially in moderate-severe cases, dilatation can further promote peristomal fibrosis, resulting in poor long-term results and in a worsening of stenosis. In this event, dilatation may be not resolutive and then represents a bridge to surgery [65].

#### Surgical treatment

Surgery represents the only definitive option in case of stenosis not responding to conservative treatment. The surgical options depend on: the underlying disease, the longitudinal extension of the stenosis, and the chance to obtain an adequate bowel length just by mobilizing the stoma stump in the peristomal field [11].

In case of superficial stenosis, a revision of stoma trephine under local anesthesia may be sufficient [85]. A cutaneous advancement flap has been also described successfully [88].

In case of a longer or deeper stenosis, an attempt in mobilizing the stoma below the fascia to obtain a longer bowel segment to resect the stenotic tract and redo the stoma is the first option [11, 84, 85]. However, if this attempt is not successful, a laparotomy with a potentially challenging adhesiolysis is required to create a new stoma, with all inherent risks related to a potential “hostile” abdomen [11].

7. Stoma bleeding

#### Definition, epidemiology, and classification

The rate of bleeding from a stoma is difficult to assess, in relation to the time of presentation and the extent of bleeding, and it ranges from 0.74% to 1.2% [63, 90]. The most frequent cause of bleeding from ostomy is from parastomal variceal bleeding. Stomal variceal hemorrhage was first described by Resnick et al. in 1968 in patients with primary sclerosing cholangitis who underwent a colonic resection and stoma creation for coexisting ulcerative colitis. In about 50% of patients, there is associated portal hypertension, and the risk of bleeding is 27% [91]. The two most common types are bowel diverting ileostomy and colostomy. Another type is urine diverting ileostomy; however, it is the least common type associated with varices. Other causes are reported to be local pathology, such as post-surgical adhesions or scarring or anatomical deformations. The varices are usually branches of the superior mesenteric vein and typically arise after about 48-month post-stoma creation but can appear as early as 5 months. The estimated mortality rate associated with stomal variceal hemorrhage is 3–4%. The point of bleeding is identified by simple inspection, and Doppler ultrasound can be useful to determine the direction of blood flow [92].

**Fig. 1:**
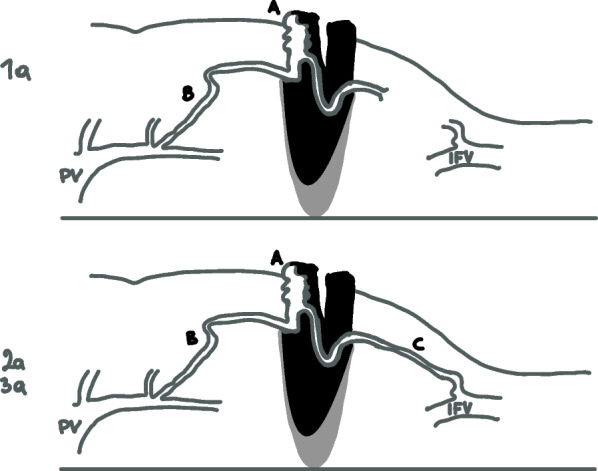
Stomal varices. Stomal varices classification revised from Saad’s article [91]. Type-a is non-occlusive and is pressure driven (oncotic), usually with some element of portosystemic collaterals (type-a2 and -a3) to decompress the higher portal pressure. **A**: parastomal varices. **B**: Afferent portal branch vein. **C**: Efferent systemic vein. *PV* portal vein. *IFV* ilio-femoral branch vein

Saad et al. described anatomy and classification of stomal varices (Fig. 1). Not all are simply due to generalized portal hypertension, but many are associated with local or segmental pathology, including adhesions, and scarring as well as the surgery-altered anatomy. The afferent stomal varices usually are mesenteric branches off the superior mesenteric vein. The involved mesenteric branch takes a sharp turn at the beginning of its extraperitoneal course and then leads to the stomal mucosa. Most cases have an indirect systemic venous drainage via multiple small anastomoses in the subcutaneous tissue of the anterior abdominal wall [93].

#### Non-operative management

The initial hemostasis maneuver can be achieved rapidly at the bedside and consists in direct pressure to the bleeding point, injection of vasoconstricting agents, cautery with silver nitrate and suturing. However, the risk of rebleeding is high unless a more definitive treatment is offered [11].

#### Operative treatment

Operative management can be divided into variceal local treatment and systemic treatment of portal hypertension.

##### Local operative treatment

Reversal or revision of the stoma is a definitive treatment, but is not often feasible due to the high perioperative surgical risk in cirrhotic patients. Mucocutaneous disconnection is reported in small cases [94].

##### Embolization

Embolization can be performed either transhepatically or percutaneously with injection of sclerosing agent but is associated with risk of bowel infarction and portal vein thrombosis, which can have a mortality rate of up to 50% [93–95].

Systemic treatment of portal hypertension includes surgical portosystemic shunt, transjugular intrahepatic portosystemic shunt (TIPS) and liver transplant [94]. When local treatment is not more effective, patient has to be addressed to a Center specialized in hepatology, hepatic surgery and liver transplantation.

## Conclusion

Stoma-related complications are frequent and require a step-up multidisciplinary management, from a conservative stoma care to surgical stoma revision. In theory, stoma reversal, when not contraindicated, is the best treatment for stoma complications no longer manageable conservatively. Application of literature evidence in clinical practice for stoma creation and an improved management of stoma-related complications could significantly improve the quality of life of patients with ostomy. However, solid evidence from the literature about their correct management is lacking, and an international consensus is needed to draw up new guidelines on this subject.

### Supplementary Information

**Additional file 1.** Detailed study protocol methods and results are available in the appendix.

## Data Availability

Methods of literature search are listed in Additional file 1.
