# Supplementary material for: Surgical management of ostomy complications: a MISSTO–WSES mapping review
Source: World J Emerg Surg. 2023 Oct 10;18:48. doi: 10.1186/s13017-023-00516-5 (PMC10563348; doi:10.1186/s13017-023-00516-5)
Supplement: Supplementary file 1 — Additional file 1. Detailed study protocol methods and results are available in the appendix. [file 13017_2023_516_MOESM1_ESM.docx]

**Appendix**

1. ***Methods and results for stoma necrosis***

A literature search in PUBMED, EMBASE, SCOPUS and COCHRANE database was performed with the keywords “stoma AND necrosis”, “ileostomy AND necrosis”, “colostomy AND necrosis”, with no language restriction and limited to the years 2011-2021. This search retrieved 884 articles. After exclusion of papers focused on pediatrics or other stoma complications (stenosis, prolapse, mucocutaneous separation, hernia, omental necrosis, retraction or pyoderma gangrenosum) a total of 21 papers were selected for the review.

1. ***Methods and results for mucocutaneous separation and stoma retraction***

A literature search in PUBMED, EMBASE, SCOPUS and COCHRANE database was performed with the keywords “stoma AND mucocutaneous separation”, “ileostomy AND mucocutaneous separation”, colostomy AND mucocutaneous separation” in the databases used for the study, with no language restriction and limited to the years 2011-2021. This search retrieved 29 articles, 14 of which were selected for the review.

A second literature search was performed with the keywords “stoma AND retraction”, “ileostomy AND retraction”, colostomy AND retraction” in the databases used for the study, with no language restriction and limited to the years 2011-2021. This search retrieved 144 articles, 30 of which were selected for the review.

1. ***Methods and results for stoma prolapse***

A literature search in PUBMED, EMBASE, SCOPUS and COCHRANE database was performed with the keywords “stoma AND prolapse”, “surgery AND stoma AND prolapse”, “surgery AND ileostomy AND prolapse”, “surgery AND colostomy AND prolapse”, with no language restriction and limited to the years 2011-2021. This search retrieved 179 articles, 20 of which were selected for the review.

1. ***Methods and results for parastomal hernia***

A first literature search in PUBMED, EMBASE, SCOPUS and COCHRANE database was performed with the keywords “parastomal AND hernia” with no language restriction and limited to the years 2011-2021.

The first selection was focused on the diagnostic methods with the following keywords: "parastomal hernia" AND classification AND (colostomy OR ileostomy). This search retrieved 17 articles, 4 of which were selected for the review. After analyzing the studies, one more article retrieved from the references of included studies, was added to the analysis, even if from 2009, since it was considered relevant for the purposes of the review.

The second part of literature review regarded the management of PSH, including the non-operative management and surgical treatment, both in emergency and elective. After a wide literature research with the keywords "parastomal hernia" AND (treatment OR repair) AND (colostomy OR ileostomy), one guideline, one position statement and some reviews were selected for a first analysis. The references included in the EHS guidelines and in the ACPGBI position statement, both from 2018, were considered as the best evidence available until that year for their rigorous scientific methods. For the subsequent years (2019-2021) only clinically relevant studies were considered, but no randomized controlled trials were found. Among the reviews, only one clearly described the research methods, the others were only narrative reviews or experts’ opinion in the form of narrative reviews, but they were considered in this study for their clear and extensive description of the management of PSH with clinically relevant articles cited in their references and for the lack of any other clinically relevant study about the management of PSH. Only one multicenter retrospective cohort study was found about the non-operative management of PSH.

1. ***Methods and results for stoma stenosis***

A literature search in PUBMED, EMBASE, SCOPUS and COCHRANE database was performed with the keywords “stoma AND stenosis”, “ostomy AND stenosis”, “ileostomy AND stenosis”, “colostomy AND stenosis” "surgical stomas AND therapeutics", "surgical stomas AND complications" were performed for the timeframe 2011-2021. This search retrieved 494 articles. After the cleaning of articles retrieved, 16 papers were included in the review. Remarkably, no one of these studies was specifically focused on this complication.

1. ***Methods and results for stoma bleeding***

A literature search in PUBMED, EMBASE, SCOPUS and COCHRANE database was performed with the keywords “stoma AND bleeding”, “ostomy AND bleeding”, “ileostomy AND bleeding”, colostomy AND bleeding”, with no language restriction and limited to the years 2011-2021. This search retrieved 17 articles, 5 of which were included in the review.
